# Aquaporin-4 autoantibodies increase vasogenic edema formation and infarct size in a rat stroke model

**DOI:** 10.1186/s12865-015-0087-y

**Published:** 2015-05-20

**Authors:** Martin Juenemann, Tobias Braun, Simone Doenges, Max Nedelmann, Clemens Mueller, Georg Bachmann, Pratibha Singh, Franz Blaes, Tibo Gerriets, Marlene Tschernatsch

**Affiliations:** Heart & Brain Research Group, Justus-Liebig-University Giessen and Kerckhoff Clinic, Benekestrasse 2-8, 61231 Bad Nauheim, Germany; Department of Neurology, Justus-Liebig-University Giessen, Klinikstrasse 33, 35392 Giessen, Germany; Department of Radiology, Kerckhoff Clinic, Benekestrasse 2-8, 61231 Bad Nauheim, Germany; Department of Pharmacology, Max-Planck-Institute for Heart and Lung Research, Ludwigstraße 43, 61231 Bad Nauheim, Germany; Department of Neurology, Kreiskrankenhaus Gummersbach, Wilhelm-Breckow-Allee 20, 51643 Gummersbach, Germany; Department of Neurology, Buergerhospital Friedberg, Ockstaedter Strasse 3-5, 61169 Friedberg, Germany

**Keywords:** Aquaporin-4, Cerebral edema, Infarct size, Neuromyelitis optica, Stroke animal model

## Abstract

**Background:**

Neuromyelitis optica (NMO) is an autoimmune disorder of the central nervous system, which is characterized by autoantibodies directed against the water channel aquaporin-4 (AQP4). As one of the main water regulators in the central nervous system, APQ4 is supposed to be involved in the dynamics of brain edema. Cerebral edema seriously affects clinical outcome after ischemic stroke; we therefore aimed to investigate whether NMO-antibodies may exert the same functional effects as an AQP4-inhibitor in-vivo in acute ischemic stroke.

**Methods:**

Sixteen male Wistar rats were randomized into two groups twice receiving either purified NMO-IgG or immune globulin from healthy controls, 24 hours and 30 minutes before middle cerebral artery occlusion (MCAO) was performed. T2-weighted MRI was carried out 24 hours after MCAO.

**Results:**

MRI-examination showed a significant increase of infarct size in relation to the cerebral hemisphere volume with NMO-IgG treated animals (27.1% ± 11.1% vs. 14.3% ± 7.2%; p < 0.05) when corrected for the space-occupying effect of vasogenic edema formation and similar results without edema correction (34.4% ± 16.4% vs. 17.5% ± 9.3%; p < 0.05). Furthermore, T2-RT revealed a significant increase in cortical brain water content of the treatment group (19.5 ms ± 9.7 ms vs. 9.2 ms ± 5.2 ms; p < 0.05).

**Conclusions:**

These results support the functional impact of NMO-antibodies and also offer an in-vivo-applicable animal model to investigate the properties of AQP4 in ischemic stroke.

## Background

Brain water homeostasis is important to prevent edema and functional disturbances in the interplay between neurons and glial cells. Brain edema contributes extraordinarily to morbidity and mortality after cerebral ischemic stroke, mainly due to its space-occupying effect [1-4]. Aquaporin-4 (AQP4) is the main glial (astrocytic) water channel in the brain and is mainly expressed at fluid-tissue borders throughout the central nervous system [5-7]. Recent data suggest that AQP4 is involved in brain edema formation as well as in edema elimination [8,9].

AQP4 has also been described as an autoantigen in neuromyelitis optica (NMO), an inflammatory disease of the central nervous system, mainly affecting the spinal cord and the optic nerve [10,11]. The clinical findings associated with AQP4-antibodies include idiopathic inflammatory demyelinating disorders summarized as “NMO-spectrum disorders”. The defining marker of these disorders is the presence of AQP4-antibodies in sera and/or cerebrospinal fluid (CSF) [12]. AQP4-antibodies are synthesized intrathecally at the disease onset and a down-regulation of AQP4 on astrocytes *in-vitro* is induced by degradation and internalization of the protein within minutes [13,14]. However, Rossi et al. recently doubted, that NMO-IgG leads to inhibition of AQP4 water permeability at all [15]. Adding complement and AQP4-antibodies to astrocytes activates the classical complement cascade leading to impaired membrane integrity of the cultured astrocytes [14,16]. Interestingly, AQP4-IgG also induced a down-regulation of excitatory amino-acid transporter 2 (EAAT2), an astrocytic glutamate transporter. Glutamate has long been known to play an important role in the pathophysiology of cerebral ischemia [16-18]. Additionally, AQP4 antibodies increase the permeability of a blood–brain-barrier model using co-cultured astrocytes and brain endothelial cells [19].

Recently, intraventricular injection of AQP4-positive IgG together with human complement has been shown to induce NMO-like inflammatory brain lesions [20], and passive transfer of NMO-IgG to EAE-rats induced an exacerbation of the disease and NMO-like lesions [21,22]. These studies produce first evidence for functional properties of AQP4-antibodies in an *in-vivo* animal model.

The involvement of AQP4 in the dynamics of brain edema and findings of *in-vitro* studies on properties of AQP4-antibodies such as the impairment of cell integrity, the promotion of excitotoxicity and increased permeability of the blood–brain-barrier, include important pathways known from the pathophysiology of ischemic stroke [23]. This leads to the assumption, that anti-AQP4 may exert also *in-vivo* effects on the extent of ischemic infarction and edema as well as on functional outcome.

In the present study, we investigated whether administration of NMO-IgG, extracted from one NMO-patient, influences the development of stroke in an MRI-based rat middle-cerebral artery occlusion model. Intravenous immune globulin can reduce infarct volume in rat models for stroke [24]; therefore, injection of immune globulin from healthy controls served as placebo-treatment in the control group. We found infarct size being almost doubled in NMO-IgG treated animals. In addition, vasogenic edema formation, as detected on quantitative T2-weighted MR-imaging, was increased in the cortex of NMO-IgG treated animals.

Our results add important information to the *in-vivo* effects of AQP4-antibodies in animal models. This model can be used to elucidate the functional properties of AQP4 in ischemic stroke in the future.

## Methods

### Animal preparation

All procedures were carried out in accordance with our institutional guidelines and the German animal protection legislation and were approved by the regional ethics committee (Regierungspraesidium Darmstadt).

Sixteen male Wistar Unilever rats (HsdCpb:WU; Harlan Winkelmann, Germany), weighing 290 to 350 g, were anesthetized with 5% isoflurane (Abbott, Wiesbaden, Germany), delivered through air at 1.0 L/min for two minutes. Anesthesia was maintained with 2-3% isoflurane delivered through air at 0.6 L/min during surgery and MR imaging. During the surgical procedure and MR measurements, body temperature was monitored with a rectal probe and maintained at 37°C by external heating.

### Middle cerebral artery occlusion and reperfusion

Middle cerebral artery occlusion (MCAO) was performed as described previously [24,25]. Briefly, the right common (CCA), internal (ICA) and external carotid artery (ECA) were exposed through a midline incision of the neck and the ECA was ligated with a 4–0 suture. A 4–0 silicone-coated nylon suture with a thermically rounded tip was introduced through an arteriotomy of the CCA. The occluder was advanced carefully into the ICA 17.0 to 21.0 mm beyond the carotid bifurcation until a mild elastic resistance indicated the tip was properly lodged in the anterior cerebral artery and thus blocked blood flow to the MCA. Then the occluder was fixed in place with a 4–0 suture. Ninety minutes after MCAO, the nylon suture was drawn from the CCA for reperfusion of the brain. Then the animals were allowed to recover from anaesthesia in individual cages with free access to food and water for the remaining survival time of 24 h.

### MR imaging

The animals were fixed in a body restrainer with a tooth-bar and a cone shaped head holder and were placed in an MRI spectrometer (Bruker PharmaScan 7.0 T, 16 cm, Ettlingen, Germany). Respiratory rate was monitored by a pressure probe placed between the restrainer and the animal’s thorax. Anesthesia was maintained with isoflurane delivered through air at 0.6 L/min. The isoflurane concentration was varied between 2.0 and 3.0% to keep the respiratory rate between 35 and 45/min. Temperature was monitored using a rectal probe and maintained at 37°C by a thermostatically regulated water flow system during the entire imaging protocol [24,25].

The MRI-tomograph operates at 300.51 MHz for ^1^H-imaging and is equipped with a 300mT/m self-shielding gradient system. The linear polarized volume resonator (diameter 60 mm) was tuned and matched manually.

Localizer images were acquired using a spin-echo sequence. RARE sequences (20 contiguous slices, 1 mm thickness, TR = 2500 ms, TE = 41.8 ms) were used to verify symmetric positioning and were repeated after correction of slice-angulation, if necessary.

### T2-Weighted Imaging (T2WI)

A Carr-Purcell-Meiboom-Gill spin echo imaging sequence was used to map lesion and hemisphere volumes. Eight contiguous coronal slices with a thickness of 2 mm (gap 0 mm) were acquired (FOV 37 × 37 mm, matrix 512 × 256, TR 3833.5 msec, 12 echoes: TE 18–216 msec (∆TE 18 msec), TA 16.25 minutes, NEX 1).

T2-relaxation time (T2RT) was measured in regions of interest within the center of the ischemic area on all slices displaying ischemic lesions and a corresponding position on the contralateral hemisphere. The difference in T2RT between the ischemic and unaffected hemispheres was calculated.

Computer aided planimetric assessment of ischemic lesion volumes and hemispheric volumes were performed by two blinded investigators experienced in experimental stroke MRI. Ipsilateral and contralateral hemispheric volume and lesion volume on T2WI were determined with the image analysis software Image J 1.25 s (National Institutes of Health, USA). After optimal adjustment of contrast, the edges of the hemispheres were traced manually on each slice, using neuroanatomic landmarks. The edges of the hyperintense ischemic lesions were traced manually in a similar fashion. The areas were then summed and multiplied by the slice thickness to calculate volumes. Lesion volumes were calculated with and without edema correction and expressed as percent of the hemispheric volume as described previously [26]:

$$ \%\mathrm{HLVuc} = \mathrm{L}\mathrm{V}/\left(\left(\mathrm{H}\mathrm{V}\mathrm{c} + \mathrm{H}\mathrm{V}\mathrm{i}\right)/2\right)*100\Big) $$

$$ \%\mathrm{HLVec} = \left(\mathrm{H}\mathrm{V}{\mathrm{c}}^2 + \mathrm{L}\mathrm{V}*\left(\mathrm{H}\mathrm{V}\mathrm{c} + \mathrm{H}\mathrm{V}\mathrm{i}\right)-\mathrm{H}\mathrm{V}{\mathrm{i}}^2\right)/\left(\mathrm{H}\mathrm{V}\mathrm{c}*\left(\mathrm{H}\mathrm{V}\mathrm{c} + \mathrm{H}\mathrm{V}\mathrm{i}\right)\right)*100 $$

(%HLVuc = percent hemispheric lesion volume – not corrected for edema; %HLVec = percent hemispheric lesion volume – corrected for edema; HVc = volume of the contralateral hemisphere; HVi = volume of the ipsilateral hemisphere; LV = lesion volume)

Midline shift (MLS) -quantification was performed on T2-weighted images where the position of the third ventricle could be determined clearly in all animals. The distance between the outer border of the cortex and the middle of the third ventricle was measured from the ipsilateral (A) and contralateral (B) side. Measurements were performed at the level of maximum lateral displacement of the ventricle. MLS was calculated using the following equation [4,27]:

$$ \mathrm{M}\mathrm{L}\mathrm{S} = \left(\mathrm{A}-\mathrm{B}\right)/2 $$

### Purification of immune globulin

Plasma from one male NMO-patient aged 41, AQP4-IgG ratio 70.79 in serum (antibody measured by radioimmunoprecipitation with a ratio <10 being negative) was diluted with glycine buffer (0.1 M, pH 9) and applied to a protein G column (HiTrap - GE) that binds exclusively IgG. The IgG-fraction was eluted by changing the pH from 9 to 2.7. The IgG-concentration was determined by nephelometry (Boehring). IgG-fractions were dialyzed against PBS to eliminate the glycine and increase the pH up to 7.4 and diluted with PBS to 9.5 g/L.

### Functional testing

Neurological evaluation was performed prior to anesthesia and 24 hours after induction of ischemia. We applied a neurological score with ten different sensorimotor and coordinative items, as described by Nedelmann et al. [28]. Furthermore, animals were placed on a rotarod that was continuously accelerated from 0 rpm to 30 rpm. The maximum speed the animals tolerated without falling off of the device was recorded [29].

### Experimental protocol

Sixteen animals were randomized into two groups, receiving either NMO-IgG (9.5 g/L) or immune globulin from a pooled sample of 10 healthy controls (control-IgG) as placebo. Two mL of immune globulin were twice injected intravenously: 24 hours and 30 minutes before MCAO.

Twenty-four hours after induction of ischemia, the animals were evaluated clinically, subjected to MR-imaging and then decapitated while under deep anesthesia. The brains were quickly removed from the skull and inspected to detect side-effects such as subarachnoid hemorrhage.

### Statistical analysis

Data are presented as mean ± standard deviation. Group differences were tested using either Mann–Whitney u-test or Student’s t-test where applicable. A p-value <0.05 was considered statistically significant.

## Results

Three animals had to be excluded from this study according to our predefined exclusion criteria [25]: One animal (control-IgG group) developed subarachnoid hemorrhage, as detected by postmortem inspection, two animals (one control-IgG group, and one NMO-IgG group) did not develop an ischemic MCA territory stroke, most likely due to inappropriate insertion depth of the occluder. All excluded animals were replaced.

The remaining animals survived and completed the study protocol. Injections of NMO-IgG and control-IgG were well tolerated by all animals without overt side effects.

Twenty-four hours after ischemia and reperfusion, all animals displayed clinical signs of MCA territory stroke. Clinical evaluation indicated a trend towards more severe neurological deficits in NMO-IgG treated rats (score 42.1 ± 11.1) as compared to control-IgG treated rats (37.1 ± 10.7). The difference, however, was not statistically significant (p = 0.391; u-test). Rotarod test performance was likewise comparable between both groups (p = 0.450; u-test).

**Figure 1:**
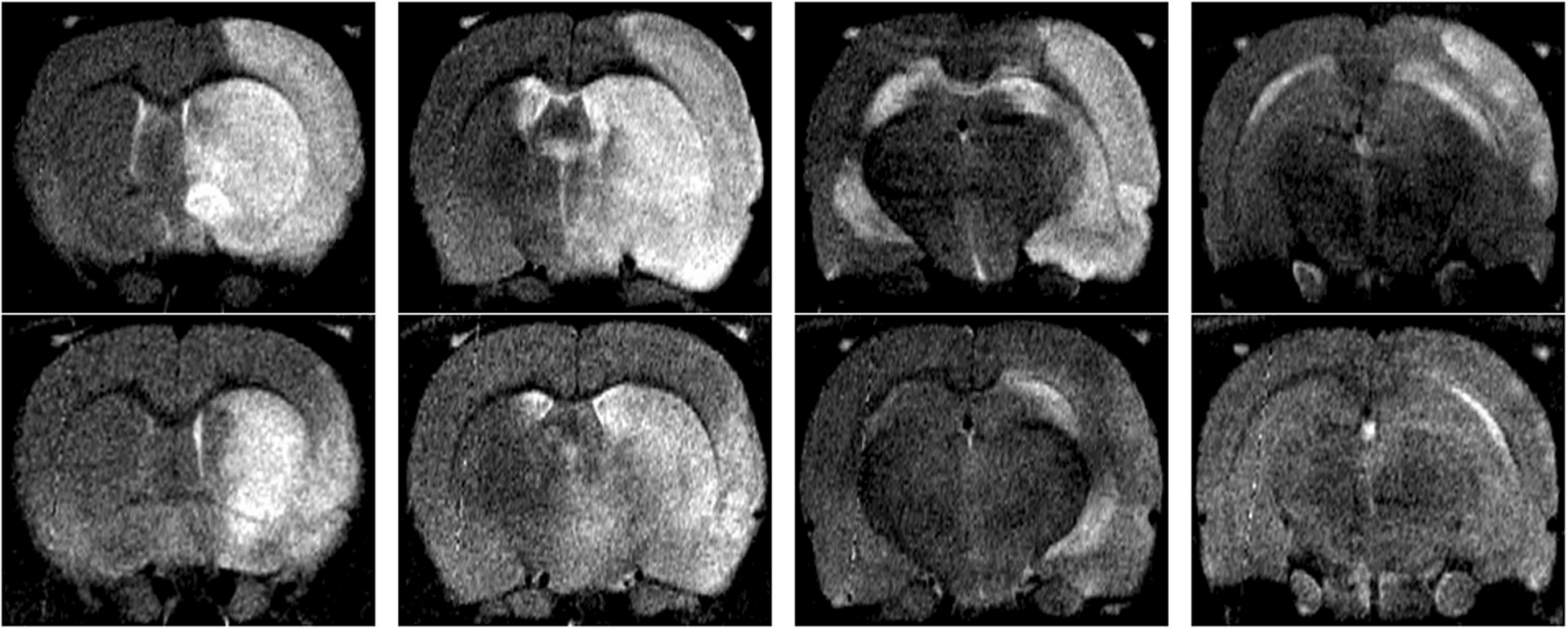
T2-weighted MRI. Representative examples of right-hemispherical ischemia in T2-weighted MRI after 24 h (contiguous slices 2–5): 1^st^ row NMO-IgG, 2^nd^ row control-IgG.

**Figure 2:**
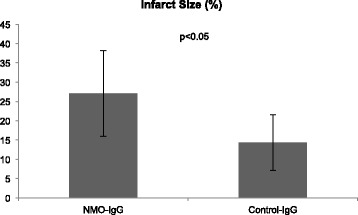
Infarct size. Treatment with NMO-IgG caused a significant increase in ischemic lesion volume compared to control-IgG (p < 0.05; expressed in percent of the affected hemisphere).

Infarct size, as determined by MRI and corrected for the space-occupying effect of vasogenic edema formation, was significantly increased in NMO-IgG treated animals (27.1% ± 11.1%) as compared to control-IgG treated rats (14.3% ± 7.2%; p = 0.026; t-test) (Figures 1 and 2). Infarct size quantification without edema correction revealed similar results (34.4% ± 16.4% vs. 17.5% ± 9.3%; p = 0.031; t-test).

**Figure 3:**
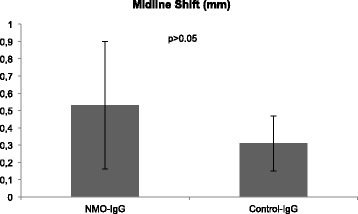
Midline shift. NMO-IgG-treatment leads to a more pronounced midline-shift as compared to control-IgG (p > 0.05).

A more pronounced midline shift was also detectable in NMO-IgG treated animals. However, this was not statistically significant (0.53 mm ± 0.37 mm vs. 0.31 mm ± 0.16 mm; p = 0.174; t-test) (Figure 3).

**Figure 4:**
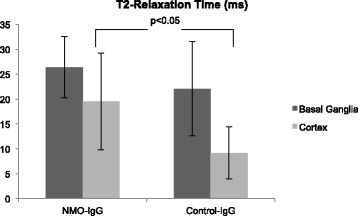
Edema formation within the basal ganglia and the cortex. T2-relaxation time, a parameter that correlates closely with vasogenic edema formation, was comparable between NMO-IgG and control-IgG treated animals if measured within the basal ganglia (p > 0.05). Vasogenic edema formation within the cortex was significantly increased in animals treated with NMO-IgG as compared to control-IgG (p < 0.05).

Quantification of the brain water content by T2-RT measurement showed different results for the cortex and the basal ganglia. In basal ganglia regions no significant difference in T2-RT could be detected (NMO-IgG: 26.4 ms ± 6.1 ms; control-IgG: 22.1 ms ± 9.5 ms; p = 0.328) (Figure 4). Within the cortex, however, NMO-IgG treatment resulted in a statistically significant increase in brain water content (19.5 ms ± 9.7 ms) as compared to rats treated with control-IgG (9.2 ms ± 5.2 ms; p = 0.045; t-test) (Figure 4).

## Discussion

With the present study we show that NMO-IgG has *in-vivo* effects in a stroke animal model, and perturbs brain water homeostasis. Treatment of rats with NMO-IgG prior to MCA-occlusion almost doubles infarct size and significantly increases cortical vasogenic edema formation.

A possible explanation for the larger infarct size might be that the application of immune globulin, per se, reduces infarct size in the control-IgG treated group. Recently, this mechanism has been described for intravenous immune globulin (IvIg), and also that it is mediated by inhibition of complement [24,30]. Thus, the control group (control-IgG) would benefit from this effect. However, since the NMO-IgG-fraction contains a normal amount of total IgG, the protective effects would be present here as well.

AQP4-IgG has recently been identified as a marker for Neuromyelitis optica, a disorder with demyelination of optic nerve and cervical spinal cord [14]. *In-vitro* observations showed that AQP4-IgG is able to induce a rapid internalization of AQP4 from the cell surface. Since these water pores are involved in the formation and elimination of vasogenic brain edema, application of AQP4-IgG might affect edema formation and brain swelling in acute ischemic stroke. Unfortunately, injection of this antibody dramatically increased edema formation in our rat stroke model.

Brain swelling due to cerebral edema can be attributed to both, cytotoxic and vasogenic brain edema, that often coexist or condition each other [31]. Evidence exists that the role of APQ4 differs between these two forms [31,32]. With respect to cytotoxic edema, where ionic imbalance results in water influx into the cell within minutes after ischemia onset, AQP4 - deficiency seems to promote edema reduction: AQP4 knock-out mice with ischemic stroke showed improved neurological outcome and reduced cerebral edema within the first 24 h after MCAO. However, compared to our study that included temporary MCAO for ninety minutes, Manley at al. chose a rodent model of permanent MCAO for 24 hours. Further more, the absolute reduction of tissue damage could not be determined, because the relation of edema in infarcted and non-infarcted regions was not known [8]. Vasogenic edema of the extracellular space is ascribed to leakage of a defective blood–brain barrier. Studies on models of vasogenic brain edema, including intraparenchymal fluid infusion, focal cortical freeze injury and tumor cell implantation in AQP4 knock-out mice showed increased elevations of intracranial pressure, increased brain water and worse neurological outcome, suggesting that AQP4 facilitates transcellular water movement and thereby clearance of vasogenic edema [33].

The promotion of vasogenic edema formation, displayed by a prolonged T2-RT, in our experiment could explain the dramatic increase in infarct size in the NMO-IgG group. Since the space-occupying effect of brain swelling is known to impair regional cerebral blood flow in the border zone of the evolving ischemic lesion - particularly within the cortex - this could explain the increase of lesion size [24,34,35]. We have previously shown that elimination of the space-occupying effect of edema formation (brought about by bilateral craniectomy prior to stroke onset) can reduce infarct size by 50%. This finding indicates that the space-occupying effect of vasogenic edema formation might be responsible for half of the ischemic damage in large territorial stroke [3]. Interestingly, T2-RT was generally longer in the basal ganglia compared to the cortex and statistically significant differences in vasogenic edema were only found in cortical areas (Figure 4). This finding may be attributed to on one hand worse vascular collateralization within basal ganglia and on the other hand to an inhomogeneous distribution of AQP4 in the brain: A study on cultured astrocytes isolated from rat cortex and striatum reasoned, that AQP4 is expressed at higher levels within the cortex compared to the striatum [36].

Another - or additional - explanation for the neurotoxic effect of AQP4-IgG is related to excitotoxicity caused by the release of glutamate during the early phase of the ischemic cascade [18]. Hinson et al. demonstrated that AQP4-IgG impairs glutamate transport by down-regulating the glutamate transporter EAAT2 along with the internalization of AQP4 [14,16]. EAAT2 is responsible for about 90% of total glutamate uptake [37]. Up-regulation of EAAT2 has been shown to induce ischemic tolerance in focal cerebral ischemia, and moreover, down-regulation of EAAT2 led to an increased infarct volume in a rat ischemia-reperfusion model [38]. Glutamate induced excitotoxicity can be further aggravated by the reversal of glutamate transporters under conditions of energy failure, i.e. stroke, where the electrochemical gradient of Na+/K+ collapses due to a decreased availability of ATP and leads to a glutamate efflux from astrocytes [39-41].

Approaches toward an animal model using AQP4-IgG necessarily involved a break-down of the blood–brain-barrier. Intravenous administration of AQP4-IgG alone did not exhibit any effects to the so treated animals [20-22]. Only intraventricular application of AQP4-IgG together with human complement, or treatment of EAE-rats with AQP4-IgG, was able to produce pathogenic effects *in-vivo*. In our model, NMO-IgG induced changes in the brain edema after an ischemic stroke. This suggests that not only inflammatory but also ischemic break down of the blood–brain-barrier results in effective targeting of AQP4-IgG.

## Conclusion

Taken together, the present study indicates that human AQP4-IgG strongly influences vasogenic edema formation and infarct size in our MRI-based rat model of focal cerebral ischemia and reperfusion. Unfortunately, the antibody leads to an increase of edema and infarct size and is therefore not suitable for clinical application. However, this animal model is eligible to further study the properties of AQP4 in ischemic stroke *in-vivo*.
